# Two-dimensional-Ti_3_C_2_ magnetic nanocomposite for targeted cancer chemotherapy

**DOI:** 10.3389/fbioe.2023.1097631

**Published:** 2023-01-25

**Authors:** Mahdieh Darroudi, Seyedeh Elnaz Nazari, Maryam Karimzadeh, Fereshteh Asgharzadeh, Nima Khalili-Tanha, Seyyedeh Zahra Asghari, Sara Ranjbari, Fatemeh Babaei, Majid Rezayi, Majid Khazaei

**Affiliations:** ^1^ Department of Physiology, Faculty of Medicine, Mashhad University of Medical Science, Mashhad, Iran; ^2^ Department of Medical Biotechnology and Nanotechnology, School of Science, Mashhad University of Medical Science, Mashhad, Iran; ^3^ Department of Electrical and Computer Engineering, University of Central Florida, Orlando, FL, United states; ^4^ Chemical Engineering Department, Faculty of Engineering, Ferdowsi University of Mashhad, Mashhad, Iran; ^5^ Metabolic Syndrome Research Centre, Mashhad University of Medical Science, Mashhad, Iran; ^6^ Department of Microbiology and Virology, Faculty of Medicine, Mashhad University of Medical Sciences, Mashhad, Iran

**Keywords:** drug delivery, cervix cancer, pH-responsive, magnetic nanocomposite, *in-vivo*, stimuli drug release

## Abstract

**Introduction:** Cervical cancer is the leading cause of cancer-related death in women, so novel therapeutic approaches are needed to improve the effectiveness of current therapies or extend their activity. In recent decades, graphene analogs, such as Mxene, an emerging class of two-dimensional (2D) graphene analogs, have been drawing considerable attention based on their intrinsic physicochemical properties and performance as potential candidates for tumor therapy, particularly for therapeutic purposes. Here we explored the targeted drug delivery in cervical cancer in *in vivo* model. Mxene-based nanocarriers are not able to be precisely controlled in cancer treatment.

**Method:** To solve this problem, the titanium carbide-magnetic core-shell nanocarrier (Ti_3_C_2_-Fe_3_O_4_@SiO_2_-FA) is also developed to provide synergetic anticancer with magnetic controlling ability along with pH-responsive drug release. A xenograft model of the cervix was used to investigate the effects of Cisplatin alone, or in combination with Ti_3_C_2_@FA and Ti_3_C_2_@ Fe_3_O_4_@SiO_2_-FA, on tumor growth following histological staining for evaluation of necrosis.

**Result and Discussion:** A significant tumor-growth suppression effect is shown when the Ti_3_C_2_-Fe_3_O_4_@SiO_2_-FA nanocarrier is magnetically controlled Cisplatin drug release. It reveals a synergistic therapeutic efficacy used in conjunction with pharmaceuticals (*p* < .001). According to the *in vivo* study, the Ti_3_C_2_@FA@Cisplatin nanocomposite exhibits less tumor growth than the drug alone or Ti_3_C_2_@FA@Cisplatin *via* increasing necrosis effect (*p* < .001). Through this study, Mxene nanosheets are expanded for biomedical applications, not only through the fabrication of biocompatible magnetic Mxene nanocomposite but also through the development of functionalization strategies that enable the magnetic Ti_3_C_2_ nanocomposite to load high levels of Cisplatin for cervical cancer treatment (242.5%). Hence, Ti_3_C_2_-Fe_3_O_4_@SiO_2_-FA nanocarriers would be promising candidates to improve cancer treatment efficiency.

## 1 Introduction

There are several different types of cancer, but cervical cancer is the one that has emerged as the primary cause of death among females. The number of cervical cancer cases diagnosed is estimated to be around 500,000 annually (Jasrotia et al., 2022). Based on their histology, cervical cancers are generally classified as squamous cell carcinoma, adenocarcinoma, and adenosquamous carcinoma (Jiang et al., 2020). The Human papillomavirus (HPV) is believed to cause 90% of cervical cancer cases (Pokhriyal et al., 2019). Traditional cancer treatments, such as surgery, radiotherapy, ablation, and chemotherapy, are often unsatisfactory because they involve invasive procedures, high recurrence rates, and side effects (Siegel et al., 2019). During the last decade, medical nanotechnology has been driving the design of new intelligent nanosystems that can respond to the pathological environment of tumor tissue with physical-morphological modifications. Nanotechnology-based therapeutics are gaining increasing attention in cancer treatment due to their multiple advantages, including a low level of invasiveness and few side-effects compared to other clinical treatments (Yu et al., 2022). In terms of clinical use, however, some limitations remain, including considering the composition and structure of nano agents, the complex physiological system, and their interactions rather than their therapeutic aspects. According to the reductionist viewpoint, the therapeutic route for nanoparticle-based therapies entails the delivery of nanoparticles to the tumor site, the treatment of the tumor with exogenous excitations, and the expulsion of nanoparticles from the body over time slowly (Liu et al., 2022).

On the other hand, it has been demonstrated that organic nanosystems possess a high potential for turning conceivable solutions to current therapeutic and diagnostic challenges into options that are more efficient and effective (Chen et al., 2021; Li et al., 2021). It has gained significant accomplishments in the fight against viruses, cardiovascular diseases, cancer, etc. (Qiu et al., 2022). Organic nanocarriers are especially appealing in cancer therapies due to their biocompatibility, enhanced cell permeability, high content of payloads, and selectivity for tumor accumulation (Zhang et al., 2021). Organic nanosystems are currently being developed based on biodegradable polymers and non-biodegradable polymers (Yu et al., 2021). Due to the absence of chronic toxic and inflammatory reactions, biodegradable organic nanocarriers are superior to their non-biodegradable counterparts (Blasi, 2019). A number of studies have demonstrated that drug carriers can significantly increase drug accumulation in specific organs and cells (Ulbrich et al., 2016), thus enabling the delivery of drugs to areas that require therapeutic effects, such as in tumor matrixes and/or in cancer cells, through controlled release (or activation). (Pecorino, 2012). Furthermore, it is possible to limit the toxicity of a drug by selective activation, which would reduce or eliminate any adverse effects and invasiveness (Nestler and Lüscher, 2019). In addition, several nanomaterials have been demonstrated to possess unique therapeutic potential, such as two-dimensional nanomaterials (Gong et al., 2017b; 2017a; Darroudi et al., 2022).

Therapeutic nanomedicine extensively explored the possibility of two-dimensional (2D) nanosheets being the wonder material of this era of science (Chen et al., 2015; Ranjbari et al., 2022), which benefited from their unique physiochemical properties and nanostructures, including their unique ultrathin nanostructure and associated desirable physiochemical and biological properties (Li X. et al., 2008; Chen et al., 2016b; Liu et al., 2018c). There are numerous 2D nanosystems, including graphene (Huang et al., 2019; Liu et al., 2021; Koutsioukis et al., 2022), black phosphorus (Qian et al., 2017; Wang et al., 2017), metals and metal oxides (Tang et al., 2014; Sun et al., 2018; Li et al., 2019), and transition metal dichalcogenides (TMDCs) (Chou et al., 2013; Shi et al., 2020), which have been applied in a variety of applications, including molecular imaging, drug/gene delivery, biosensing, photothermal/photodynamic therapy, antibacterial activity, and even tissue engineering (Khafaji et al., 2019; Zhang et al., 2020). An emerging component of 2D nanomaterials is Mxene, a new and emerging compound that represents a large group of transition metal carbides as well as carbonitrides (Naguib et al., 2011; Bai et al., 2021; Shen et al., 2021). Mxene is prepared through the ablation of A elements from ternary-layered carbides of MAX phases in which M represents a transition metal carbide, A represents an element of the A group, and X represents a C or N element (Karlsson et al., 2015; Wang et al., 2015; Ferrara et al., 2021). Furthermore, in addition to the most extensively investigated applications of Mxene in the energy storage field, we, along with other researchers, recently demonstrated that ultrathin titanium carbide (Ti_3_C_2_) nanosheets could be intrinsically engineered to be highly effective in theragnostic and tumor therapy (Xuan et al., 2016; Li R. et al., 2017; Song et al., 2021). The development of 2D Ti_3_C_2_ Mxene for antibacterial, fluorescent imaging, and biosensing applications has opened a new research frontier for using Mxene in biomedical applications. In recent years, therapeutic nanomedicine has proven effective at treating various diseases by combining diagnostic imaging and therapeutic functionality (Lee et al., 2015; Liu et al., 2015; Chen et al., 2017). In order to achieve the specific functionalities and performances, it would be highly beneficial if this 2D Ti_3_C_2_ Mxene could be integrated with other functional moieties; however, it is still a challenge and unattainable as of yet (Zhang et al., 2018; Yang et al., 2021). In order to fully functionalize Ti_3_C_2_ Mxene, the functional moieties must be directly decorated onto the surface of nanosheets while maintaining the intrinsic Mxene properties and framework (Zhang et al., 2022). Taking this into account, we present the development of magnetic Ti_3_C_2_ Mxene for cancer therapy applications by directly coating Ti_3_C_2_ Mxene with magnetic iron oxide nanoparticles. Based on the specific surface chemistry and versatile properties of magnetic iron oxide nanoparticles, Mxene nanosheets provide an ideal surface for the application (Liu et al., 2018b; Ma et al., 2021). Despite this, Mxene-based nanosheets led to a lack of controllability as nanocarriers and have a low drug loading content, so many nano vehicles can’t remain in the tumor site continuously due to blood circulation, resulting in the inevitable damage to normal tissues as well as a decline in anticancer effectiveness (Xing et al., 2018).

It is still a challenge to improve the drug-loading capability of Ti_3_C_2_-based nanocarriers for cancer treatment while providing controllability to confine them inside cancer cells. It has been demonstrated in previous studies that superparamagnetic nanomaterials can control the movement of nanoscale drug carriers (Shahzad et al., 2018; Yang et al., 2018). Using cobalt ferrite/graphene oxide (CoFe_2_O_4_/Go), Wang et al. revealed that the nano platform could be controlled due to its magnetic properties (Wang et al., 2016). There have been a few studies in which magnetic material has been introduced into drug delivery systems to demonstrate high levels of drug-loading ability (Chan et al., 2017). According to the above studies, by combining magnetic materials with Ti_3_C_2_-based nanocarriers, it might be possible to resolve the dilemma.

When a magnetic nanomaterial was introduced into Ti_3_C_2_ nanosheets to form a drug nanocarrier, the magnetic nanocarriers would be confined in cancer cells under an external magnetic field, and then nanocarriers would be efficiently contacted by cancer cells. In response to endogenous or exogenous stimulation, the nanocarrier releases the anticancer drug (Cisplatin), resulting in more effective responsive therapy for localized tumor eradication. According to our knowledge, a controllable nanocarrier for combating cancer cells has not yet been achieved by combining Ti_3_C_2_ nanosheets with magnetic core shells. Therefore, for the first time, we investigated the effect of combining Ti_3_C_2_ nanosheets with magnetic core shells on the suppression of cervical cancer tumor growth.

## 2 Material and methods

### 2.1 Materials

Ti_3_AlC_2_ (powder, 200-meshes), HF, HCl (36%, w/w, purity >98%), TMAOH (25 wt% in water), AlCl_3_ 6H_2_O, and Folic acid, sodium dodecyl sulfate (SDS), phosphate buffer saline (PBS), high glucose cell culture medium, and a tetrazolium-based assay were purchased from Sigma-Aldrich. No further treatment was applied to the chemicals unless otherwise stated. An aqueous solution of monobasic potassium phosphate (KH_2_PO_4_) and dibasic potassium phosphate (K_2_HPO_4_) was utilized to produce PBS at pH 7. We used ultrapure water throughout all experiments.

### 2.2 Characterizations

Bruker diffractometers (PW1730) were used to carry out X-ray diffraction (XRD) with Cu Kα radiation (λ = 1.5406 Å). In order to characterize the morphology of the samples, transmission electron microscopes (TEM, Philips EM208S 100 KV) and field emission scanning electron microscopes (FESEMs, Hitachi, Japan) were used. To measure absorbance from 200 to 800 nm, Uv/Vis spectrometer (Perkin Elmer Lambda 25) was used. In addition, a JASCO FT-IR-460 spectrometer was utilized to obtain Fourier transform infrared spectroscopy (FT-IR) in the 400 to 4,000 cm^−1^ range. Using vibrating sample magnetometers (VSM), magnetic nanocomposites were measured at Mahamax, Tehran, Iran).

### 2.3 Fabrication of titanium carbide (Ti_3_C_2_) nanosheets

A sample of 1 g of Ti_3_AlC_2_ powder was etched in a solution containing 1 g of LiF and 0.3 g of AlCl_3_ 6H_2_O for 3 days at room temperature with 10 mL of HCl (9 M) (Liu P. et al., 2018). The etching materials were centrifuged several times before being dispersed in 10 mL of TMAOH for 3 days, followed by centrifugation and washing to remove the intercalated Ti_3_C_2_. In order to prepare a colloidal suspension of Ti_3_C_2_ nanosheets, a clay-like solid was dispersed in water for hours under bath sonication, then the supernatant was centrifuged at 7,000 rpm, and then a freeze-drier was used to collect the dried samples.

### 2.4 Fabrication of Ti_3_C_2_-Fe_3_O_4_@SiO_2_-FA

As part of the preparation of Ti_3_C_2_-Fe_3_O_4_@SiO_2_ nanocomposites, 50.0 mg of Fe_3_O_4_@SiO_2_ nanoparticles synthesized hydrothermally (Darroudi et al., 2020; Ghasemi et al., 2021) was dispersed ultrasonically in 20 mL deionized water, and 100.0 mg of Ti_3_C_2_ nanosheets were dispersed in 80 mL deionized water and stirred for 30 min (Dai et al., 2017). In both solutions, ultra-sonification was performed for 120 min under an Ar atmosphere. After filtering the suspension to obtain the Ti_3_C_2_-Fe_3_O_4_@SiO_2_ nanocomposite, it was dried in a vacuum overnight at 60°C. Afterward, 100.0 mg of the prepared Ti_3_C_2_-Fe_3_O_4_@SiO_2_ and 0.5 g of folic acid were dispersed in 60 mL of deoxygenated water for 1 h with ultrasound. Then, the reaction system was kept in an Ar atmosphere in an oil bath at 60 C for 4 h. Finally, the obtained Ti_3_C_2_-Fe_3_O_4_@SiO_2_-FA was washed with water and dried with a freeze-dryer.

### 2.5 Fabrication of Ti_3_C_2_@FA-Cisplatin

As part of the preparation of Ti_3_C_2_@FA nanocomposites, 5.0 mg of folic acid (FA) functionalized polymer was dispersed ultrasonically in 10 mL deionized water, and 100.0 mg of Ti_3_C_2_ nanosheets were dispersed in 50 mL deoxygenated water and stirred for 30 min, and then mixed through sonicating for extra 90 min under an Ar atmosphere. After filtering the suspension to obtain the Ti_3_C_2_@FA nanocomposite, it was dried in a vacuum overnight at 60°C. Finally, the obtained Ti_3_C_2_@FA was washed with water and dried using freeze-dryer.

### 2.6 Drug loading and release of Ti_3_C_2_-Fe_3_O_4_@SiO_2_-FA-Cisplatin

#### 2.6.1 Drug adsorption study

As part of the cisplatin adsorption study, the following nanocarriers (Ti_3_C_2_-Fe_3_O_4_@SiO_2_-FA and Ti_3_C_2_@ FA) (1 mL) loaded formulations were mixed separately with various masses of Cisplatin (0.5, 1, 1.5, 2, 3 mg) of Cisplatin in 20 mL of Phosphate-buffered saline solution (PBS) under an ice-cooled dark environment (Jermy et al., 2019; 2021). After overnight stirring and sonication at room temperature, the solution mixture was centrifuged at 8,000 rpm for 10 min. Then, the product was collected and washed with 15 mL of standard saline solution. Based on the equation, the amount of Cisplatin adsorption was calculated using UV visible spectroscopy at 293 nm.

The supernatants were centrifuged for different intervals of time (0, 0.5, 1.5, 2.5, and 6 h). A UV-vis spectrometer was used to measure the absorbance of supernatants and then to calculate the Cisplatin concentration based on a standard calibration curve based on the characteristic absorbance peak of Cisplatin (293 nm) in 2 mL.

Drug loading of Cisplatin: (w/w%) = (the weight of final Cisplatin in Ti_3_C_2_-Fe_3_O_4_@SiO_2_-FA-Cisplatin)/(weight of initial Ti_3_C_2_-Fe_3_O_4_@SiO_2_-FA) × 100%.

#### 2.6.2 Drug release study

The cumulative cisplatin release was studied using the prepared nanoformulations Ti_3_C_2_-Fe_3_O_4_@SiO_2_-FA-Cisplatin and Ti_3_C_2_@FA -Cisplatin (Ramezani Farani et al., 2022). Drug delivery was performed by immersing 10 mg of drug formulations in 20 mL of phosphate-buffered saline (PBS) at pH 4.5 and pH 7.4 (for selected samples). The release condition was performed at a constant temperature of 37°C. A specific volume of solution (2 mL) was removed at regular intervals, replaced with fresh PBS solution, and analyzed using UV-visible spectrometry. In order to calculate the weight of the final Cisplatin, the amount of the original Cisplatin was subtracted from the concentration of the loaded nanocarrier. A standard calibration curve was used to calculate Cisplatin concentration based on absorbance at 293 nm. As a means of designing the standard calibration curve, six different concentrations of Cisplatin solution (1, 5, 10, 25, 50, 100 μg/mL) were tested by UV–Vis spectrophotometer at the characteristic peak of Cisplatin absorbance (293 nm).

### 2.7 *In-vivo* intraperitoneal administration under an external magnetic field

In order to examine Ti_3_C_2_-Fe_3_O_4_@SiO_2_-FA-Cisplatin’s anticancer properties, it was examined on the cervix tumor model. The C57BL/6 mice (25–30 g) were purchased from the Laboratory Animal Center of Mashhad University of Medical Sciences (MUMS), Mashhad, Iran. The Ethical Committee approved animal experimentation protocols of the Experimental Animal Center at MUMS, Mashhad, Iran. Standard food and water were provided during the experiments, and mice were housed in a laboratory environment. The TC1 cells (murine cervix cancer cell lines) were obtained from Pastour Institute (Tehran, Iran) and cultured in RPMI-1640 medium with 10% heat-inactivated FBS and 1% streptomycin at 5% CO_2_ at 37°C. 2.5 × 10^6^ TC1 cells per 100 mL were injected in the left flank region of the mouse subcutaneously injected into mice, and approximately 2 weeks later, the tumor size reached 80–100 mm^3^ (Ghaemi et al., 2012). The participants were divided into four groups randomly according to the following instructions (n = 6 in each group): I. Control group (Untreated group), II. Cisplatin as a standard regimen for the treatment of cervical cancer (administered twice at a 3-day interval using 5 mg/kg, intraperitoneally; IP), III. Ti_3_C_2_-Fe_3_O_4_@SiO_2_-FA-Cisplatin (administered twice at a 3-day interval using 5 mg/kg, IP), and IV. Ti_3_C_2_@Cisplatin (administered twice at a 3-day interval using 5 mg/kg, IP), followed by applying an external circular magnet (10 mm by 10 mm, 0.4 T surface field strength). The tumor size of the animals was measured once every other day. In order to determine the tumor volume (V), the formula V = AB^2^/2 was used, where A is the primary axis length and B is the minor axis length (Ghaemi et al., 2012). The cervical tumors of each mouse were removed on day 18 to undergo further investigation by hematoxylin and eosin staining (H&E).

### 2.8 *In-vitro* studies

#### 2.8.1 Growth inhibition studies

Cisplatin, Ti_3_C_2_@FA@Cisplatin, and Ti_3_C_2_-Fe_3_O_4_@SiO_2_-FA-Cisplatin have been evaluated for their growth inhibitory properties after 24-h and 72-h treatments. After seeding cells (5 × 10^6^) in 96-well plates, they were grown for 24 and 72 h. In the next step, cells were treated with 10 µL concentrations of Cisplatin (1–1,500 µg), Ti_3_C_2_@FA@Cisplatin (1–1,500 µg), and Ti_3_C_2_-Fe_3_O_4_@SiO_2_-FA-Cisplatin (1–1,500 µg) in the total volume of 200 μL cells per well. As previously described, the plates were then processed (Hashemzadeh et al., 2022).

#### 2.8.2 Formation of multicellular spheroids

A total of 200 µL of RPMI-1640 and GlutaMAX-I (1:1) in agarose-coated 96-well plates were seeded at a density of 5 × 10^4^ cells per well and later treated with Cisplatin, Ti_3_C_2_@FA@Cisplatin, and Ti_3_C_2_-Fe_3_O_4_@SiO_2_-FA-Cisplatin in the total volume of 200 µL per plate. On a Leica Microsystems GmbH inverted phase contrast microscope (Wetzlar, Germany), the cell connections and cytotoxic effects were assessed over 3 days. Images of spheroid sizes were analyzed with ImageJ ver. 1.8.0–112 (National Institutes of Health, Bethesda, MD, United States) (Hashemzadeh et al., 2022).

### 2.9 Statistical analysis

The results of all experiments are expressed as the mean and standard error. One-way ANOVA using LSD *post hoc* test was used to analyze the significance of the results using SPSS software (SPSS Inc., Armonk, NY, USA). Statistical significance was determined by *p* .05.

## 3 Result and discussion

### 3.1 Fabrication and characterization of the Ti_3_C_2_-Fe_3_O_4_@SiO_2_-FA-Cisplatin composite nanosheet

**FIGURE 1:**
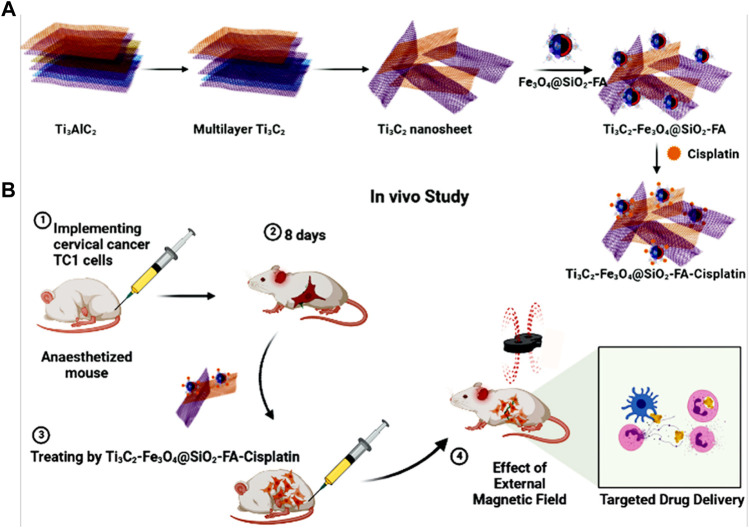
**(A)** Fabrication of Ti_3_C_2_-Fe_3_O_4_@SiO_2_-FA-Cisplatin, **(B)** Cisplatin targeting for cervical cancer delivery *in vivo* study.

Through sonication treatment, multi-layer Ti_3_C_2_ was converted into ultrathin Mxene nanosheets (Figure 1A). After this step, magnetic nanoscale Mxene sheets exhibited a large planar structure and good dispersity, which are ideal for biomedical applications (Soleymaniha et al., 2019). An HCl/LiF etchant was used initially to remove the Al layer from Ti_3_AlC_2_. In this manner, the Ti_3_AlC_2_ can be exfoliated using HCl/LiF etching, but the resulting multi-layer Ti_3_C_2_ nanosheets remain close together, exhibiting large particle sizes, which is incompatible with the demands of biomedicine (Naguib et al., 2011). Due to this, ultrasonication was used to complete the separation process and simultaneously reduce particle size. To increase controllability, magnetic nanoparticles were intercalated into Mxene layers to form heterostructures, thereby ensuring the confinement of Ti_3_C_2_ nanosheets. The Ti_3_C_2_-Fe_3_O_4_@SiO_2_-FA nanocarrier can store the charged functional groups of the anticancer drug (Cisplatin) owing to adequate hydroxyl groups on the surface (Figure 1A). For chemotherapy purposes, Cisplatin can be targeted released from nanocarriers under inner or external stimulation. As a result of their nanoscale size, Ti_3_C_2_-Fe_3_O_4_@SiO_2_-FA nanocarrier would circulate easily within blood vessels and could passively penetrate tumor cells (Li X. et al., 2017). The Ti_3_C_2_-Fe_3_O_4_@SiO_2_-FA nanocarrier can be reached cancerous cells using an external magnetic field and would not leave the cancer cells with blood circulation (Figure 1B).

**FIGURE 2:**
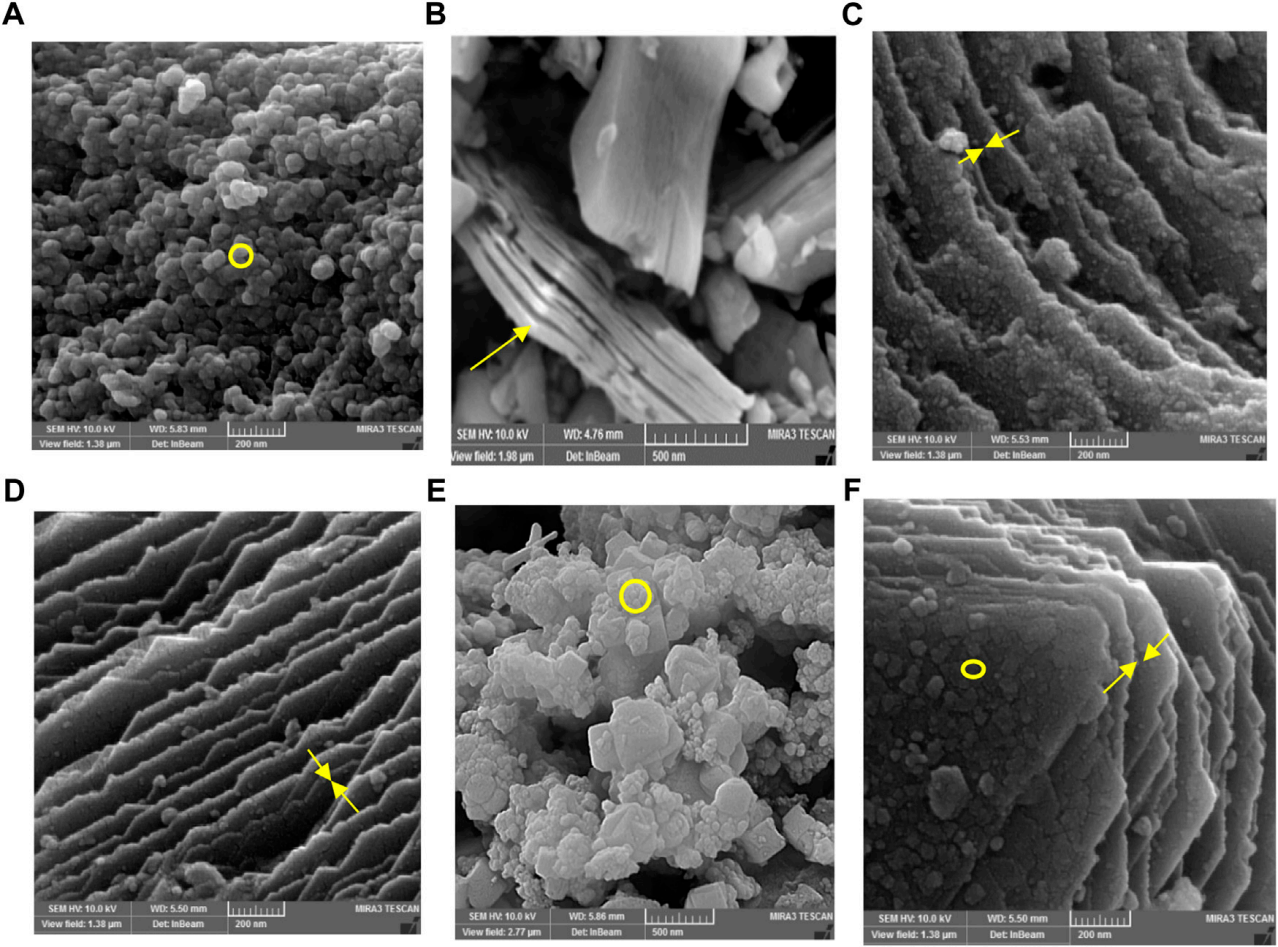
FESEM analysis of **(A)** Fe_3_O_4_@SiO_2_, **(B)** MAX phase, **(C)** Ti_3_C_2_-Fe_3_O_4_@SiO_2_-FA, **(D)** Ti_3_C_2_@FA, and **(E)** Ti_3_C_2_-Fe_3_O_4_@SiO_2_-FA-Cisplatin, and **(F)** Ti_3_C_2_@FA-Cisplatin.

**FIGURE 3:**
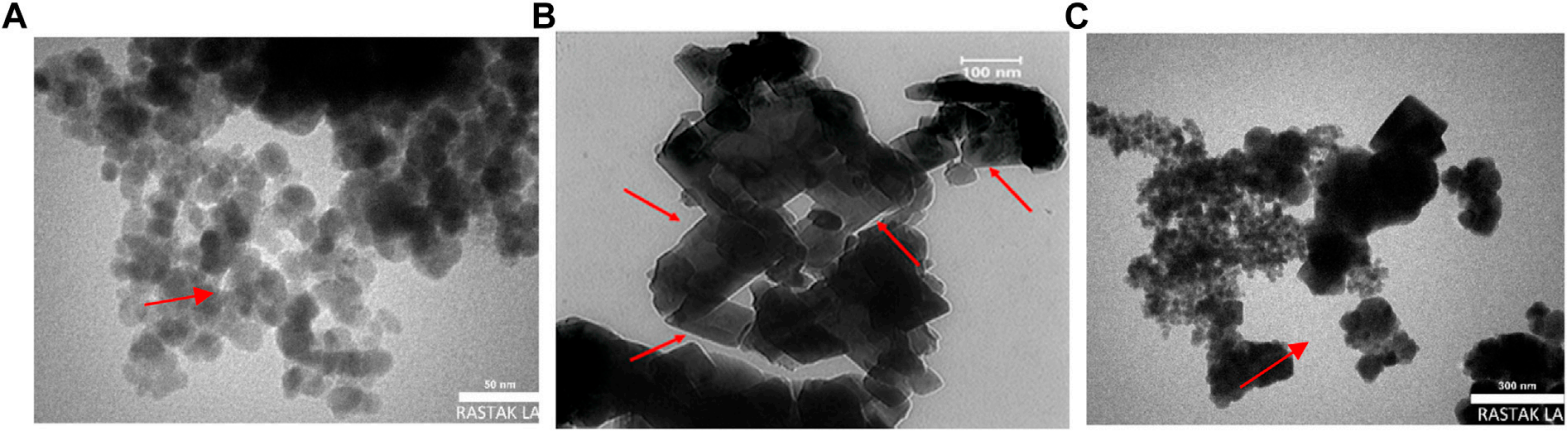
TEM analysis of **(A)** Fe_3_O_4_@SiO_2_, **(B)** Ti_3_C_2_@FA, and **(C)** Ti_3_C_2_-Fe_3_O_4_@SiO_2_-FA.

**FIGURE 4:**
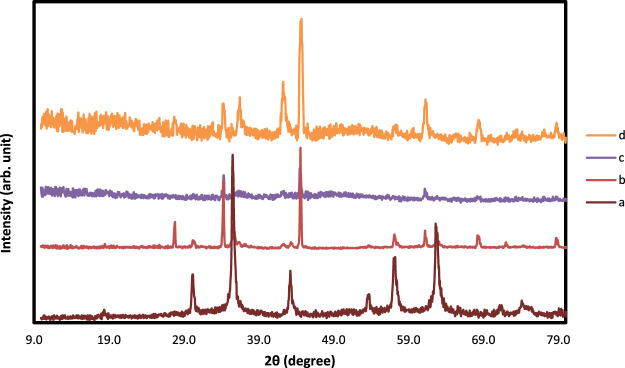
XRD analysis of **(A)** Fe_3_O_4_@SiO2, **(B)** Ti_3_C_2_-Fe_3_O_4_@SiO_2_-FA, **(C)** Ti_3_C_2_-Fe_3_O_4_@SiO_2_-FA-Cisplatin, and **(D)** Ti_3_C_2_@FA.

The morphology and structure of constructed nanocomposites are characterized using TEM and FESEM images. As illustrated in Figure 2A, the Mxene Ti_3_C_2_ nanosheet, after etching the Al layer and following intercalation by TMAOH, the FESEM image depicted that closely pack layer structure of MAX phase exfoliating into nano size thickness sheets. Also, the EDAX analysis of the etched sample confirmed the surface modification by Al ion. TEM image also revealed that the titanium carbide nanosheets have an average lateral size of approximately 35 nm (Figure 3A). Furthermore, the formation of Mxene nanosheets is confirmed by XRD analysis, in which no peak at 38̊ was exhibited through intercalation, while the characteristic peaks from stacked Mxene nanosheets appeared. As shown in Figure 2C, the Brightfield FESEM image of Ti_3_C_2_-Fe_3_O_4_@SiO_2_-FA composite nanosheets revealed a small Fe_3_O_4_@SiO_2_ magnetic core-shell with an average size of approximately 31.3 nm on the surface of Ti_3_C_2_ Mxene nanosheets with a thickness of ∼31 nm. It is clear from the TEM image (Figure 3A) that the core shells of Fe_3_O_4_@SiO_2_ species are in core-shell morphology. As shown in Figure 2D, the TEM and SEM images of functionalized Ti_3_C_2_ Mxene by FA had a surface of sheet-like morphology with a thickness of around ∼29.3 nm (Figure 2B). According to FESEM images of Ti_3_C_2_@FA nanosheet surface become roughened and covered by some spheres composed of Ti_3_C_2_@FA nanocomposite. Furthermore, the vanishing or decreasing peaks in the 30–40-degree range confirm the 2D structure of functionalized Ti_3_C_2_ nanosheets (Figure 4D).

Along with characterized nanocarrier, it exhibited a multi-layer structure of constructed Ti_3_C_2_-Fe_3_O_4_@SiO_2_-FA-Cisplatin nanosheets that Fe_3_O_4_@SiO_2_ component in core-shell morphology is firmly attached to the surface of Ti_3_C_2_ nanosheets in the average size of 27.2 nm on the sheets with thickness 30.5 nm (Figure 2E). In Figure 2F, FESEM images of Ti_3_C_2_@FA and the multi-layer Ti_3_C_2_ are shown, where a typical accordion-like structure of the multi-layer Ti_3_C_2_ can be observed with a thickness of approximately 38.5 nm.

**FIGURE 5:**
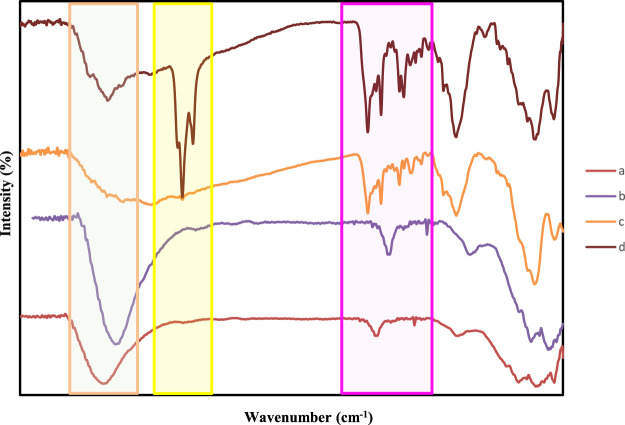
FT-IR spectra of **(A)** Fe_3_O_4_@SiO_2_, **(B)** Ti_3_C_2_@FA **(C)** Ti_3_C_2_-Fe_3_O_4_@SiO_2_-FA, and **(D)** Ti_3_C_2_-Fe_3_O_4_@SiO_2_-FA-Cisplatin.

The FESEM of MAX phase exhibited in Figure 2, confirmed the accordion-like structure of Ti_3_AlC_2_ structure, in which, through the ultrasonic process, the weak covalent bonds between the multi-layer Ti_3_C_2_ nanosheets are destroyed, confirmed through TEM image (Figure 3B). The EDAX analysis also confirmed the presence of Ti, Al, and C elements in the structure of the MAX phase (Figure 5A). Overall, the TEM image of Ti_3_C_2_ nanosheets revealed further details about their morphology and structure (Figure 3), which are approximately 29–41 nm in transverse dimension. Further, TEM results suggest that Ti_3_C_2_ nanosheets could be used as drug delivery nanocarriers due to their small thickness and size, which enhances blood circulation (Wang et al., 2013). An XRD pattern for Ti_3_C_2_ nanosheets, shown in Figure 4, demonstrates the etching of Al layers from Ti_3_AlC_2_ by using LiF/HCl etchant successfully removed Al layers from Ti_3_AlC_2_ through the most intense XRD peak (104) of Ti_3_AlC_2_ (2θ ≈ 39°). As a consequence of the introduction of lithium and hydroxyl ions into the Ti_3_C_2_ layers, the main peak (002) of Ti_3_AlC_2_ moved from 10.5° to 9.2°, since the distance between adjacent Ti_3_C_2_ layers in Ti_3_C_2_-Fe_3_O_4_@SiO_2_-FA, Ti_3_C_2_-Fe_3_O_4_@SiO_2_-FA-Cisplatin, and Ti_3_C_2_@FA is augmented by the presence of these ions (Wang et al., 2021). The aqueous dispersion of Ti_3_C_2_ nanosheets appears dark-colored, with the incident light scattered by colloidal nanosheets. The formation of Ti_3_C_2_ is also confirmed by X-ray diffraction (XRD).

**FIGURE 6:**
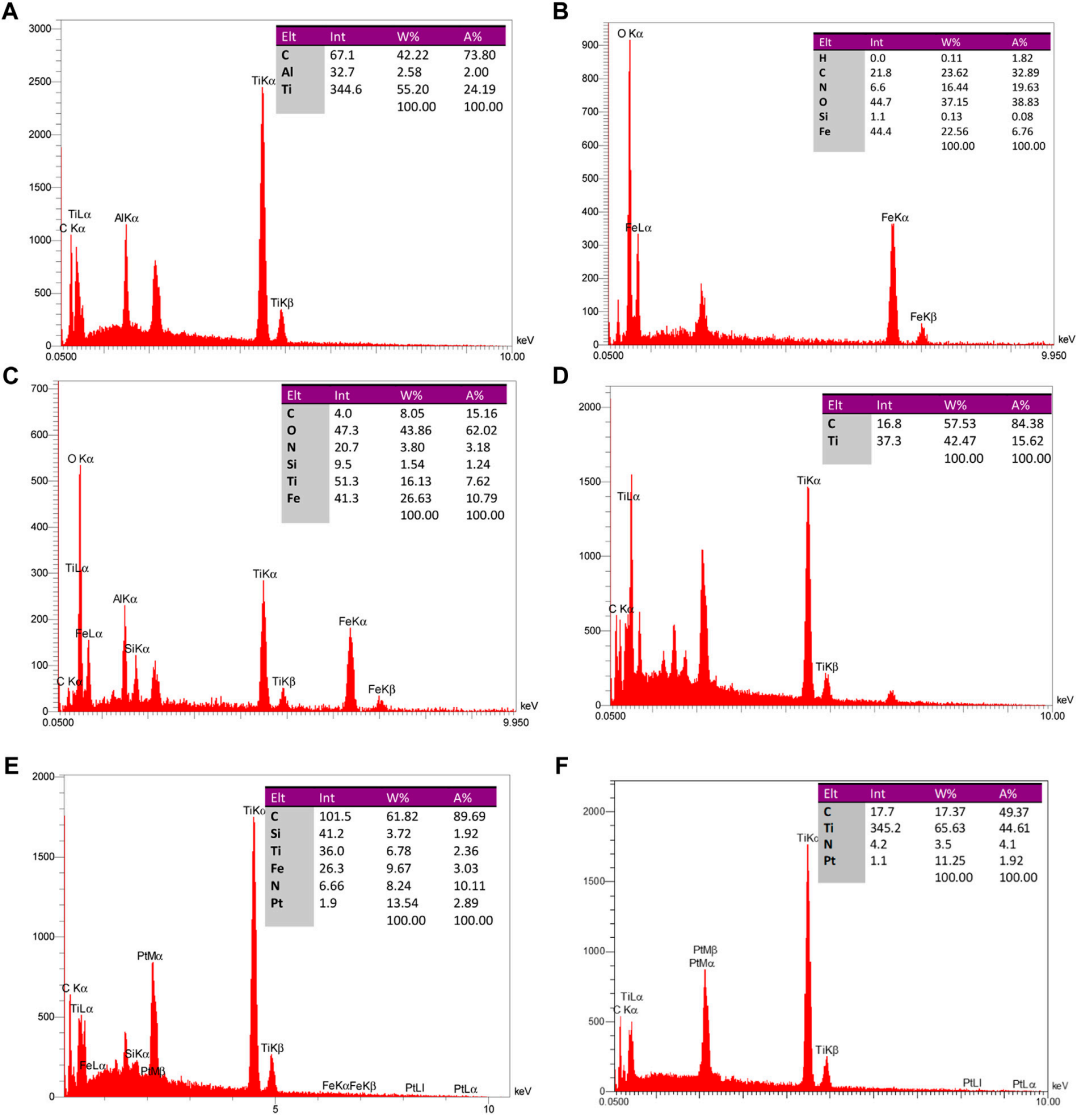
EDAX analysis of particle constituents of **(A)** Ti_3_AlC_2,_ **(B)** Fe_3_O_4_@SiO_2_, **(C)** Ti_3_C_2_-Fe_3_O_4_@SiO_2_-FA, **(D)** Ti_3_C_2_@FA, **(E)** Ti_3_C_2_-Fe_3_O_4_@SiO_2_-FA-Cisplatin, and **(F)** Ti_3_C_2_@FA-Cisplatin.

As indicated by previous studies (Ma et al., 2021), Ti_3_C_2_ MXene exhibited negatively charged oxygen-containing groups, which could absorb the positively charged Fe_3_O_4_@SiO_2_ (Zhang et al., 2022). Analysis of this reaction procedure was conducted using Fourier transform infrared (FTIR). Following iron oxide and silane core shells, Fe_3_O_4_@SiO_2_, Ti_3_C_2_-Fe_3_O_4_@SiO_2_-FA, and Ti_3_C_2_-Fe_3_O_4_@SiO_2_-FA-Cisplatin FIT-IR profiles are illustrated in Figure 6. FT-IR spectroscopy was used to verify the chemical modifications. According to the spectra analysis of Ti_3_C_2_-Fe_3_O_4_@SiO_2_-FA, the FTIR spectrum showed C–H stretch at 2,820–2,950 cm^−1^, and C–O stretch at 1,335–1,192 cm^−1^. In the range between two peaks of 1,620 and 1,440 cm^−1^, strong bond vibrations are observed for NH and NH_2_. The NH_3_^+^ stretch is a wide peak at 3,230–2,600 cm^−1^. In addition to C=O stretch at 1,694 cm^−1^ and C–O stretch at 1,340–1,180 cm^−1^, C=C stretch in aromatic compounds was also found at 1,600–1,450 cm^−1^ in the FA molecule (Figure 6). The FTIR spectra of Ti_3_C_2_-Fe_3_O_4_@SiO_2_-FA exhibited a strong band at 1,626 cm^−1^ attributing to the N-H vibration of FA. Furthermore, the FTIR spectrum of Ti_3_C_2_-Magnetic nanosheets showed a new peak at 575 cm^−1^, correlated with Fe-O stretching vibration of Fe_3_O_4_, demonstrating Fe_3_O_4_ deposition on Ti_3_C_2_ nanosheets in comparison with the FTIR spectrum of Ti_3_C_2_ nanosheets. Ti_3_C_2_-Magnetic NPs exhibit a significant reduction in the FTIR band associated with hydroxyl group at 3,411 cm^−1^ and carbonyl group at 1,690 cm^−1^ (Figure 6). The Ti_3_C_2_-Fe_3_O_4_@SiO_2_-FA-Cisplatin showed weak amine bands, suggesting that the NH_2_ was conjugated with the OH groups of the nanocomposites and Folic acid towards the Cisplatin. Moreover, the bending vibration at 1,650 cm^−1^ indicates that Cisplatin has been functionalized, as well as the reaction between the Folic acid and Pt (II) complex.

In order to examine the presence of synthetic magnetic core shells and nanosheets, SEM and EDAX were used. As shown in Figure 5, magnetic nanoparticles with an average width of 27–40 nm have a microstructure.

The distribution maps of Ti, Fe, Si, C, and O elements on Ti_3_C_2_-Fe_3_O_4_@SiO_2_-FA composite nanosheets confirmed the coexistence of Ti and Fe elements and the uniform distribution of Fe_3_O_4_@SiO_2_ on the surface. An EDAX analysis of Ti_3_C_2_-Fe_3_O_4_@SiO_2_-FA composite nanosheets (Figure 6B) shows that Ti, Fe, and Si elements are present, indicating that Fe_3_O_4_@SiO_2_ was successfully functionalized on the surface of Ti_3_C_2_ Mxene nanosheets. Furthermore, through loading Cisplatin on the nanocarrier, the existence of Pt would be exhibited in the EDAX analysis, confirming the construction of the Ti_3_C_2_-Fe_3_O_4_@SiO_2_-FA-Cisplatin and Ti_3_C_2_@FA-Cisplatin (Figures 6E, F). Insets in Figure 5 provide a list of results of the elemental analysis of Fe_3_O_4_@SiO_2_-uncoated, Ti_3_C_2_-Fe_3_O_4_@SiO_2_-FA-coated, and Ti_3_C_2_@FA-coated nanoparticles, in which 6.76%, 10.79%, and 15.62% of nanoparticles’ weights can be found to be respectively. Ti_3_C_2_-Fe_3_O_4_@SiO_2_-FA-Cisplatin contains 2.89% Pt, while Ti_3_C_2_@FA-Cisplatin contains 1.92% Pt. The elemental analysis indicates that since Fe_3_O_4_@SiO_2_ and adsorbed-Mxene on Fe_3_O_4_@SiO_2_, the remaining weight is comprised of Fe and Ti (from Fe_3_O_4_ and Ti_3_C_2_). Thus, the weight% of Fe and Ti is estimated to be 26.63% and 16.13%. The number ratio of compounds is determined to be C (8): Fe (26): Ti (16), using the mentioned weight% ratio. Therefore, 3.03:1 and 2.05:1 have been determined for the Fe_3_O_4_:Ti_3_C_2_ number and weight ratios. FT-IR analysis illustrates the existence of Ti_3_C_2_ MXene nanosheets as indicated by the elemental analysis results. Regarding the elemental analysis of Ti_3_C_2_@FA nanosheets, it should be noted that the weights% of C and Ti atoms are, respectively, determined to be 84.38% and 15.62% (Figure 6D). The C: Ti ratio is determined to be 5.41:1. In this case, C is attributed to both Ti_3_C_2_ and Folic acid coated on the nanosheets. Therefore, we conclude that the Fe_3_O_4_@SiO_2_:Ti_3_C_2_ weight% ratio is 26.53:16.13, with a 25.5% weight ratio of Fe in the total weight of the nanocarrier.

**FIGURE 7:**
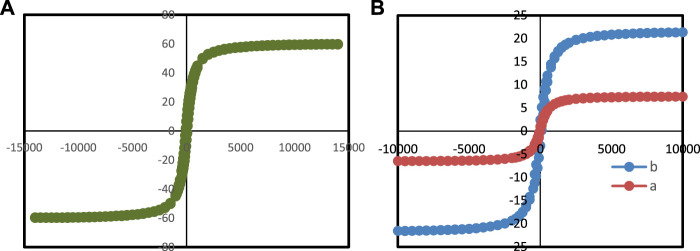
VSM analysis of **(A)** Ti_3_C_2_-Fe_3_O_4_@SiO_2_-FA, **(B)** (a) Ti_3_C_2_-Fe_3_O_4_@SiO_2_-FA-Cisplatin, and (b) Fe_3_O_4_@SiO_2_.

Due to the presence of magnetic Fe_3_O_4_ nanoparticles in Ti_3_C_2_-Magnetic composite nanosheets, the Mxene can modulate their magnetic properties by applying an external magnetic field (Figure 7), which suggests that magnetic fields can be used for further nanomedicine applications. Figure 7 shows the unique magnetic property of Ti_3_C_2_-Magnetic NPs composite nanosheets, which exhibit a saturation magnetization of 23.5 emu g^−1^.

### 3.2 The magnetic properties of Ti_3_C_2_-Fe_3_O_4_@SiO_2_-FA and Ti_3_C_2_-Fe_3_O_4_@SiO_2_-FA-cisplatin

Considering the magnetic properties of Fe_3_O_4_ core-shell nanomaterials (Gao and Wang, 2014; Oravczová et al., 2021), a magnetically controlled nanocarrier based on Ti_3_C_2_-Fe_3_O_4_@SiO_2_-FA was investigated. It is shown in Figure 7B that Fe_3_O_4_@SiO_2_, Ti_3_C_2_-Fe_3_O_4_@SiO_2_-FA, and Ti_3_C_2_-Fe_3_O_4_@SiO_2_-FA-Cisplatin nanocarriers show field-dependent magnetization curves. It was determined that Fe_3_O_4_@SiO_2_ has the highest saturation magnetization (60.0 emu/g) (Figure 7A), whereas Ti_3_C_2_-Fe_3_O_4_@SiO_2_-FA-Cisplatin nanocarrier (6.5 emu/g) exhibits significantly lower saturation magnetization than Ti_3_C_2_-Fe_3_O_4_@SiO_2_-FA (23.5 emu/g), owing to the presence of Ti_3_C_2_ nanosheets, the remanence of Ti_3_C_2_ nanocarriers is lower than the remanence of Fe_3_O_4_@SiO_2_ nanosheets (Liu et al., 2008). In conclusion, the Ti_3_C_2_-Fe_3_O_4_@SiO_2_-FA and Ti_3_C_2_-Fe_3_O_4_@SiO_2_-FA-Cisplatin nanocarriers show hysteresis loops, suggesting they could be magnetically controlled drug carriers. Furthermore, previous studies have indicated that saturation magnetizations of 16.3 emu/g would be sufficient for magnetic control (Deng et al., 2014).

### 3.3 The loading/releasing Cisplatin capability of Ti_3_C_2_-Fe_3_O_4_@SiO_2_-FA and Ti_3_C_2_@FA

**FIGURE 8:**
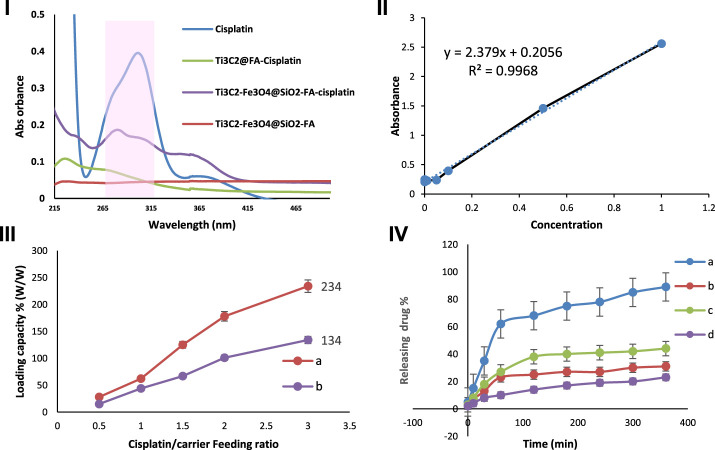
I) UV-Vis absorbance of Cisplatin, Ti_3_C_2_-Fe_3_O_4_@SiO_2_-FA, Ti_3_C_2_-Fe_3_O_4_@SiO_2_-FA-Cisplatin, and Ti_3_C_2_@ FA-Cisplatin. II) Standard calibration curve for different concentrations of Cisplatin (2.5, 5, 10, 20, 50, 100) irradiation at 293 nm, III) Cisplatin release profile of Ti_3_C_2_-Fe_3_O_4_@SiO_2_-FA and Ti_3_C_2_@FA at different drug/nanocarrier ratios (0.5, 1, 1.5, 2, 3) irradiation at 293 nm, and IV) The Cisplatin release from Ti_3_C_2_-Fe_3_O_4_@SiO_2_-FA-Cisplatin **(A, C)** and Ti_3_C_2_@-FA-Cisplatin **(B, D)** in PBS (0.1 M) solution at two pH 4.5, and 7.4, respectively (n = 3).

It has been demonstrated that Ti_3_C_2_ nanosheets (Karlsson et al., 2015) and Fe_3_O_4_ (Darroudi et al., 2020) can be further developed into drug delivery nanocarriers for cancer therapy due to the abundance of hydroxyl functional groups and their large surface areas. For evaluating the drug loading/release ability of Ti_3_C_2_-Fe_3_O_4_@SiO_2_-FA and Ti_3_C_2_@FA nanocarriers, Cisplatin, a chemotherapy drug used to combat cancer, was used as a model drug. After vigorous stirring and sonication under an Ar atmosphere, Cisplatin was loaded on the surface of Ti_3_C_2_-Fe_3_O_4_@SiO_2_-FA nanocarrier. Due to the strong electrostatic interactions between the negatively charged surface of the Ti_3_C_2_-Fe_3_O_4_@SiO_2_-FA and Ti_3_C_2_@FA nanocarriers and the positively charged Cisplatin, we achieved a high drug loading content (Liu et al., 2019). This can be seen in the shaded part of the UV–Vis spectrums (Figure 8A), where the acquired Ti_3_C_2_-Fe_3_O_4_@SiO_2_-FA and Ti_3_C_2_@FA exhibit the characteristic absorption peak of Cisplatin compared to the Ti_3_C_2_-Fe_3_O_4_@SiO_2_-FA after loading with Cisplatin. In this case, the combination between both Ti_3_C_2_-Fe_3_O_4_@SiO_2_-FA and Ti_3_C_2_@FA nanocarriers and Cisplatin would be a successful result of the interaction between the hydroxyl groups of Ti_3_C_2_-Fe_3_O_4_@SiO_2_-FA and Ti_3_C_2_@FA nanocarriers and Cisplatin (Zhao et al., 2019). Furthermore, since the constructed titanium carbide nanocarriers are negatively charged, it may offer better cell accessibility and hydrophilic properties (Chen et al., 2016a).

A UV-vis spectroscopy study was conducted to assess the capacity of the Ti_3_C_2_-Fe_3_O_4_@SiO_2_-FA and Ti_3_C_2_@FA nanocarriers to load different weight ratios of Cisplatin (0.5, 1, 1.5, 2, 3). By using a standard calibration curve for Cisplatin under 293 nm irradiation (Figure 8C), the concentration of Cisplatin was calculated and incorporated into the drug loading equation. As shown in Figures 8A–C greater ratio of Ti_3_C_2_-Fe_3_O_4_@SiO_2_-FA results in an increase in the drug loading capacity. In accordance with the equation for drug loading, the drug loading efficiency at a mass ratio of three of the Ti_3_C_2_-Fe_3_O_4_@SiO_2_-FA nanocarrier was calculated to be 244.01% based on the drug loading formula, while the Ti_3_C_2_@FA-Cisplatin loading efficiency was 134.01%. These are considerably higher than the average drug loading efficiency for single Ti_3_C_2_ nanosheets (89%) and most drug delivery nanocarriers (10%–50%) (Liu et al., 2020). It is assumed that the increase in drug loading capacity can be attributed to two factors: 1) In the case of the Ti_3_C_2_-Fe_3_O_4_@SiO_2_-FA nanocarrier, there are more electrostatic sites available for loading Cisplatin 2) The Ti_3_C_2_-Fe_3_O_4_@SiO_2_-FA nanocarrier provides sufficient surface area for Cisplatin to be loaded.

This study examined the pH-dependent drug release of Ti_3_C_2_-Fe_3_O_4_@SiO_2_-FA-Cisplatin and Ti_3_C_2_@FA-Cisplatin under several pH values (7.4 and 4.5, respectively). Figure 8D exhibited that at pH 7.4 and 4.5 during a 6-h period, 31.59%, and 89.01%, of Cisplatin are released from Ti_3_C_2_-Fe_3_O_4_@SiO_2_-FA -Cisplatin, respectively, suggests that it is more readily released in an acidic microenvironment. In comparison, Ti_3_C_2_@FA-Cisplatin exhibited a pH-responsive release profile; during 6 h, Cisplatin release was measured to be 44.31% and 23.04%, corresponding to pH 4.5 and 7.4, respectively. Due to the altered interaction between Cisplatin and Ti_3_C_2_-Fe_3_O_4_@SiO_2_-FA nanocarriers and the greater solubility of Cisplatin at lower pH values, this result can be attributed to the altered interaction between drug and Ti_3_C_2_-titanium carbide nanocarriers. Notably, at pH 7.4, a large number of hydroxyl groups on the surface of Ti_3_C_2_-Fe_3_O_4_@SiO_2_-FA become deprotonated and negatively charged, in contrast to Cisplatin’s positive charge (Gong et al., 2016). The solubility and hydrophilic properties of Cisplatin increase with an increase in protonation of the amino group under a pH value of 4.5 (Du et al., 2010). Meanwhile, the hydroxyl groups on the surface of the nanocarrier Ti_3_C_2_-Fe_3_O_4_@SiO_2_-FA are protonated in this process, would result in a repulsive interaction between Cisplatin and Ti_3_C_2_-Fe_3_O_4_@SiO_2_-FA nanocarrier (Mu et al., 2019). Since Cisplatin releases from Ti_3_C_2_-Fe_3_O_4_@SiO_2_-FA-Cisplatin in an acidic environment, the pH-triggered drug release action has a significant impact.

### 3.4 *In vivo* effects of Ti_3_C_2_-Fe_3_O_4_@SiO_2_-FA-Cisplatin magnetic nanosheets and Ti_3_C_2_@Cisplatin on cervical cancer and necrosis

**FIGURE 9:**
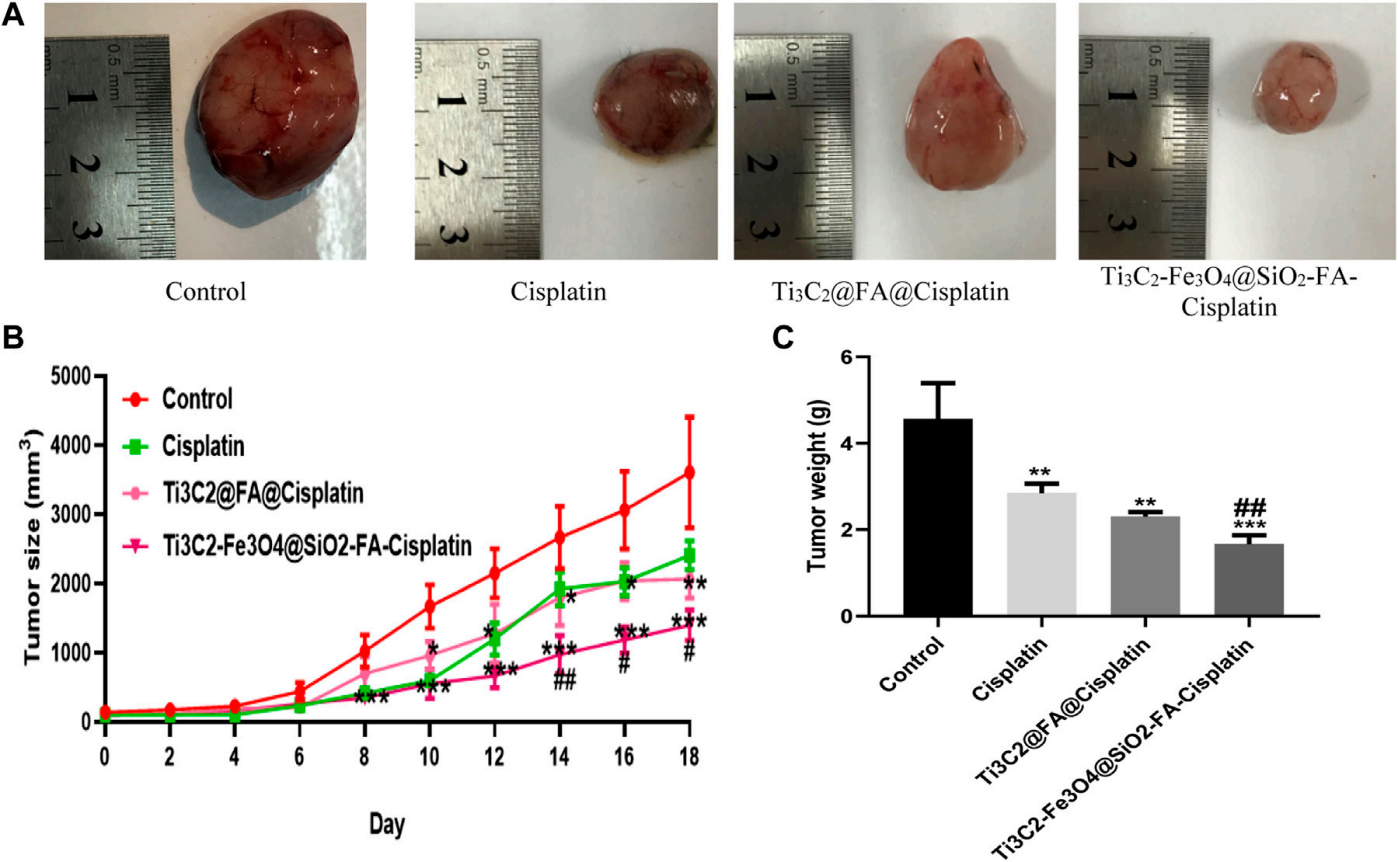
**(A)** Ti3C2-Fe3O4@SiO2-FA-Cisplatin reduces tumor size and weight in cervical cancer tumors. Effect of Ti_3_C_2_-Fe_3_O_4_@SiO_2_-FA-Cisplatin, Ti_3_C_2_@Cisplatin, and Cisplatin on tumor size **(B)** during experiment and tumor weight **(C)** in a cancerous mouse model of cervical cancer at the last of experiment.

This study aimed to determine whether Ti_3_C_2_-Fe_3_O_4_@SiO_2_-FA-Cisplatin and Ti_3_C_2_@Cisplatin inhibited the growth of cervical carcinomas in mice using a tumor model. Figure 9A in each group of mice, Ti_3_C_2_-Fe_3_O_4_@SiO_2_-FA-Cisplatin, Ti_3_C_2_@Cisplatin, and Cisplatin alone were treated, and tumor weight and size were measured. The results of *in vivo* experiments indicated a decrease in tumor size and weight in mice after treatment with Ti_3_C_2_-Fe_3_O_4_@SiO_2_-FA-Cisplatin (Figures 9B, C). This cervical cancer model shows that Ti_3_C_2_-Fe_3_O_4_@SiO_2_-FA-Cisplatin exhibits enhanced anticancer activity compared with Cisplatin alone Ti_3_C_2_@Cisplatin nanosheets, which significantly increases Cisplatin’s antitumor activity. According to the *in vivo* release profile of nanocomposite, Cisplatin alone and Ti_3_C_2_@Cisplatin nanosheets follow a one-compartment model *in vivo*. Since these magnetic nanocomposites consist of multiple compartments, they would be released sequentially, followed by the continuous release resulting from burst releases.

A significant challenge facing the treatment of cervical cancer is how to prevent the accumulation of cancer-fighting drugs in healthy tissue while improving the local accumulation of these drugs at the tumor site (Koning et al., 2010). A new therapeutic approach for treating localized cancer may be possible with nanoparticles with magnetic properties (Kumar and Mohammad, 2011). Recent research reports that a polymeric nanocapsule containing 5-Fu could treat colon cancer similarly (Li S. et al., 2008). A study by Shakeri-Zadeh et al. found that 5-Fu had an increased tendency to cause colon tumors when loaded into magnetic nanoparticles (Shakeri-Zadeh et al., 2014). In this regard, our *in vivo* experiments have shown that when Cisplatin is loaded into Mxene-magnetic nanosheets, it would have a sustained release in a pH-responsive manner, prolonged half-life, and significantly increased tumor uptake, while there was no predicted efficiency for Mxene Ti_3_C_2_@Cisplatin.

**FIGURE 10:**
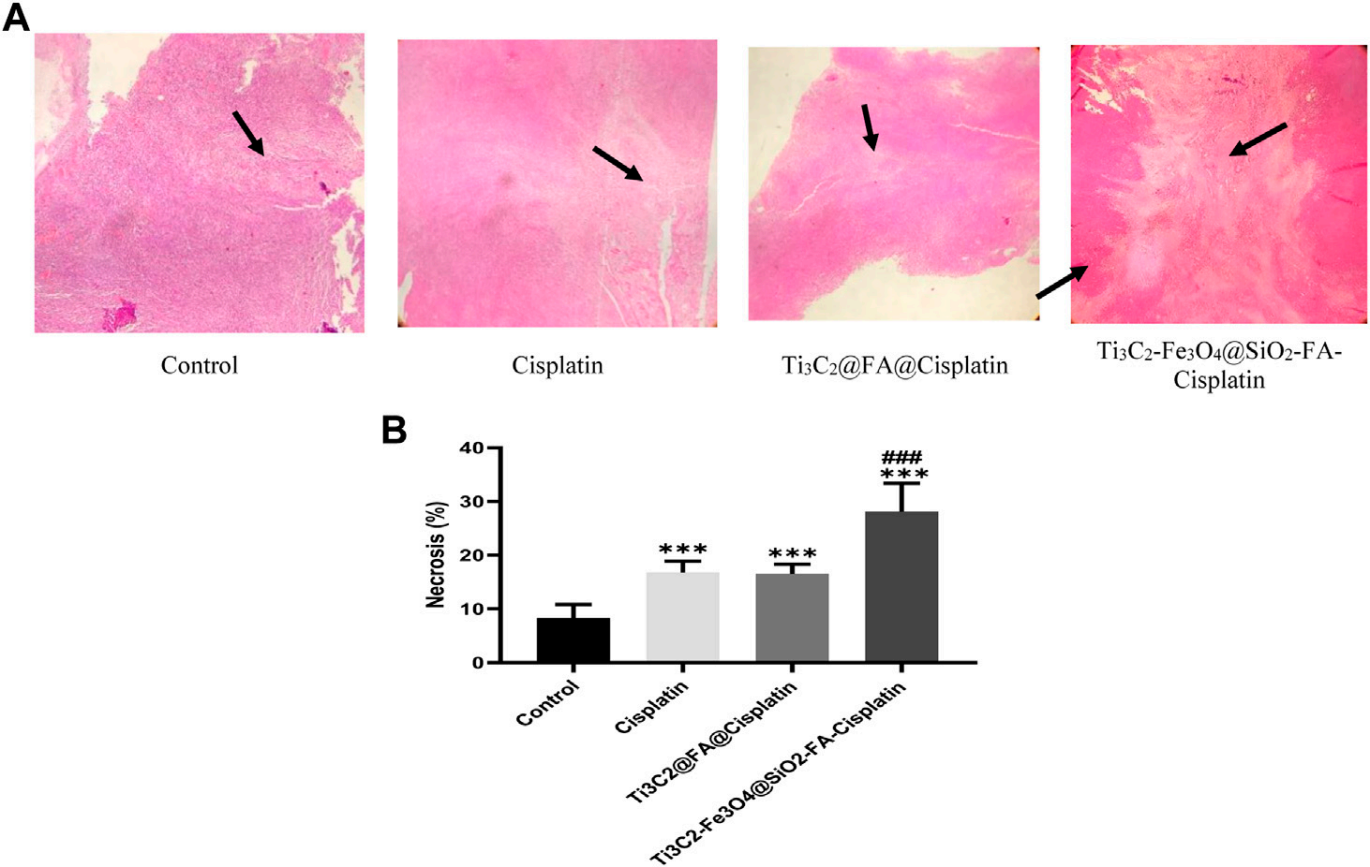
The combination of Ti_3_C_2_-Fe_3_O_4_@SiO_2_-FA-Cisplatin promotes necrosis in cervical cancer tumors compared to Ti_3_C_2_@FA@Cisplatin and Cisplatin alone. The tumor necrosis is indicated by H&E staining under a light microscope **(A)**; necrosis areas are indicated with arrows. **(B)**Using the image J software, quantify the necrotic area.

An increased area of tissue necrosis was observed in the cervix tumor after Ti_3_C_2_-Fe_3_O_4_@SiO_2_-FA-Cisplatin treatment (Figure 10A). It was revealed that Ti_3_C_2_-Fe_3_O_4_@SiO_2_-FA-Cisplatin increased percentages of necrosis in cervical tissue compared to Cisplatin alone and Ti_3_C_2_@Cisplatin as the standard chemotherapeutic regimen in cervical cancer (Figure 10B; *p* = .05). Using H&E staining, arrows indicate the necrosis area.

## 4 *In vitro* effects of Ti_3_C_2_-Fe_3_O_4_@SiO_2_-FA-Cisplatin magnetic nanosheets and Ti_3_C_2_@Cisplatin on cervical cancer

### 4.1 Ti_3_C_2_-Fe_3_O_4_@SiO_2_-FA-Cisplatin magnetic nanosheets and Ti_3_C_2_@Cisplatin inhibit cell viability

**FIGURE 11:**
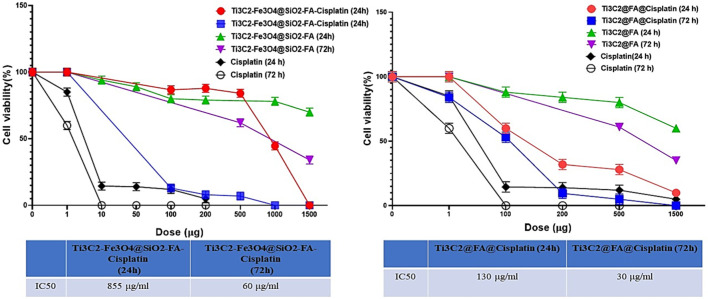
The MTT assay was used to assess cell viability after 24 h and 48 h incubation in TC1 cervix cancer cells to determine the ability of Ti_3_C_2_@FA@Cisplatin and Ti_3_C_2_-Fe_3_O_4_@SiO_2_-FA-Cisplatin to inhibit cell growth.

To examine the anti-proliferative potential of Cisplatin, Ti_3_C_2_@FA@Cisplatin, and Ti_3_C_2_-Fe_3_O_4_@SiO_2_-FA-Cisplatin, cells were exposed to the rising concentrations (0–1,500 ppm) for 24 h and 72 h. A reduction of nearly 90% in cell viability was observed at the highest concentration of free Cisplatin. In contrast, although both Ti_3_C_2_@FA@Cisplatin and Ti_3_C_2_-Fe_3_O_4_@SiO_2_-FA-Cisplatin had radical negative effects on cell viability, neither was as effective as Cisplatin. This finding also reflects in the determined IC50 values and could be attributed to variations in the drug loading/release profiles between Ti_3_C_2_-Fe_3_O_4_@SiO_2_-FA-Cisplatin and Ti_3_C_2_@FA@Cisplatin. IC50 values were found to be for Ti_3_C_2_-Fe_3_O_4_@SiO_2_-FA-Cisplatin (24 h): 855 μg/mL, (72 h): 60 μg/mL. Additionally, IC50 in terms of the amount Ti_3_C_2_@FA@Cisplatin (24 h): 130 μg/mL, (72 h): 30 μg/mL (Figure 11).

### 4.2 Cancer spheroids are inhibited by Ti_3_C_2_@FA@Cisplatin and Ti_3_C_2_-Fe_3_O_4_@SiO_2_-FA-Cisplatin

**FIGURE 12:**
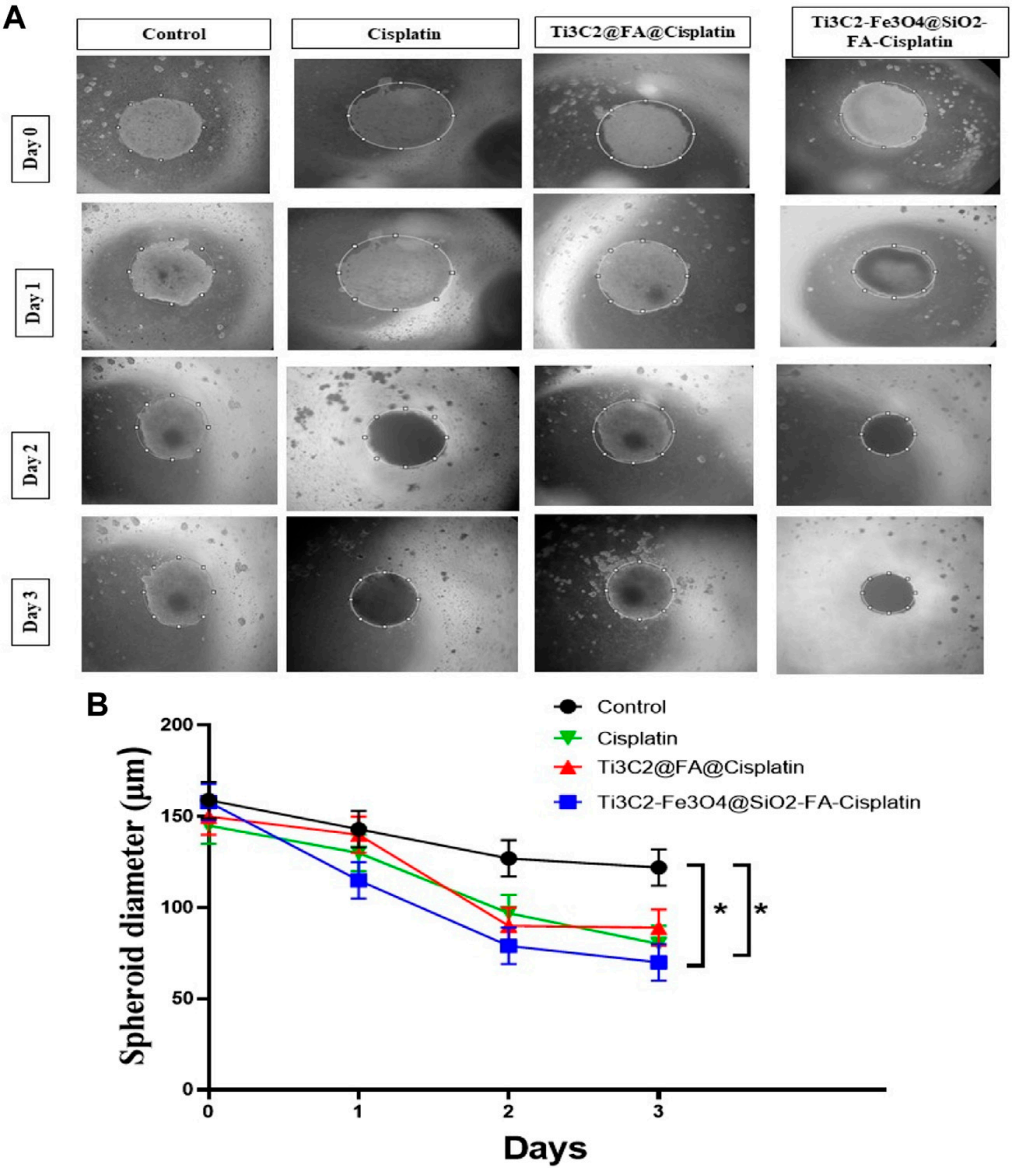
The formation of TC1, Ti_3_C_2_@FA@Cisplatin, and Ti_3_C_2_-Fe_3_O_4_@SiO_2_-FA-Cisplatin -induced spheroid cells in response to Cisplatin and Ti_3_C_2_-Fe_3_O_4_@SiO_2_-FA-Cisplatin treatments. These micrographs illustrate the effects of Cisplatin, Ti_3_C_2_@FA@Cisplatin, and Ti_3_C_2_-Fe_3_O_4_@SiO_2_-FA-Cisplatin on TC1 spheroids **(A)**. A quantitative assessment of the changes in spheroid sizes **(B)**.

In a 3-D cell structure (Figure 12), Ti_3_C_2_@FA@Cisplatin and Ti_3_C_2_-Fe_3_O_4_@SiO_2_-FA-Cisplatin were assessed for their specific anti-cancer abilities. The spheroid size of the treated group differed significantly from the control group after 3 days. Spheroid area did not change significantly after 3 days in the control group, while it significantly decreased in Cisplatin, Ti_3_C_2_@FA@Cisplatin, and Ti_3_C_2_-Fe_3_O_4_@SiO_2_-FA-Cisplatin treated groups. As a result of the loss of cell membrane integrity and the loss of viability of the core cells of Ti_3_C_2_@FA@Cisplatin and Ti_3_C_2_-Fe_3_O_4_@SiO_2_-FA-Cisplatin spheroids, the peripheral cells have already disappeared. A three-dimensional model of cervical cancer cells showed that treatments could prevent proliferation and progression, and consequently, the cancerous cells are more likely to die (Figure 12). Spheroid growth is not only significantly retarded in the presence of nanoforms, but also a corona of dead and fragmented cells is visible after approximately 3 days of treatment.

## 5 Conclusion

In conclusion, we present an efficient cancer treatment based on the magnetic functionalization of Mxene (2D Ti_3_C_2_). As a result of the in-situ growing of Mxene onto Ti_3_C_2_ Mxene surfaces, a heterostructure of Mxene-based magnetic nanoplatforms was developed. This nanoplatform can be used for synergistic therapy with pH-dependent drug release and controlled magnetic therapy for targeted effects for target therapeutic agents. In the case of Cisplatin, Ti_3_C_2_-Fe_3_O_4_@SiO_2_-FA nanocarriers demonstrated high drug loading capacities (234%) and were capable of exhibiting a drug release behavior as a result of pH stimulation. Furthermore, Ti_3_C_2_-Fe_3_O_4_@SiO_2_-FA nanocarrier could be controlled under the external magnetic field due to its magnetic properties. Finally, a first report on the multi-functionalities of Mxene, combined with Cisplatin, has been presented to demonstrate that Mxene can be elaborately engineered to fabricate magnetic nanocomposite materials using their surface chemistry and that this paradigm can be applied to a variety of therapeutic applications.

Accordingly, it was exhibited that Ti_3_C_2_@Cisplatin nanosheets do not have the anticipated efficiency in the drug delivery system. The development of magnetic Mxene-based 2D materials is expected to significantly expand the areas and potent applications, particularly in nanobiotechnology and nanomedicine of Mxene-based 2D materials. Therefore, it is anticipated that Ti_3_C_2_-Fe_3_O_4_@SiO_2_-FA nanocarriers will be an effective method for improving the cancer treatment’s efficiency.

## Data Availability

The raw data supporting the conclusions of this article will be made available by the authors, without undue reservation.
