# Supplementary material for: Two-dimensional-Ti3C2 magnetic nanocomposite for targeted cancer chemotherapy
Source: Front Bioeng Biotechnol. 2023 Jan 25;11:1097631. doi: 10.3389/fbioe.2023.1097631 (PMC9905703; doi:10.3389/fbioe.2023.1097631)
Supplement: Supplementary file 1 [file DataSheet1.docx]

Supplementary Material

**Two-Dimensional-Ti_3_C_2_ Magnetic Nanocomposite for Targeted Cancer Chemotherapy**

*Mahdieh Darroudi ^1,2, #^, Seyedeh Elnaz Nazari ^1#^, Maryam Karimzadeh ^2^, Fereshteh Asgharzadeh ^1^, Nima Khalili-Tanha ^1^, Seyyedeh Zahra Asghari ^1^, Sara Ranjbari ^3^, Fatemeh Babaei^1^, Majid Rezayi^2,4,5^*, Majid Khazaei^1,4^***

^1^ Department of Physiology, Faculty of Medicine, Mashhad University of Medical Science, Mashhad, Iran

^2^ Department of Medical Biotechnology and Nanotechnology, School of Science, Mashhad University of Medical Science, Mashhad, Iran

^3^ Chemical Engineering Department, Faculty of Engineering, Ferdowsi University of Mashhad, Mashhad, Iran

^4^ Metabolic Syndrome Research Centre, Mashhad University of Medical Science, Mashhad, Iran

^5^ Department of Microbiology and Virology, Faculty of Medicine, Mashhad University of Medical Sciences, Mashhad, Iran

**Zeta Potential**

The particle size and its distribution of the dispersed magnetic drug nanoparticles were measured by the Dynamic Light Scattering (DLS).

The results achieved from dynamic light scattering (DLS) analysis (Figure S1) showed that after preparation of Ti_3_C_2_Tx@Fe_3_O_4_@SiO_2_-FA, the size range has changed to 32.2 ± 8.4 nm which approves the presence of a new layer on MNPs. Drug loading also caused NPs to be 31.3 ± 6 nm in diameter with a negative charge.

**
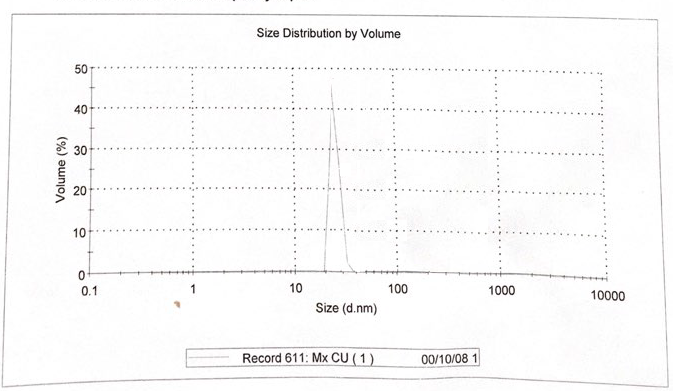
**


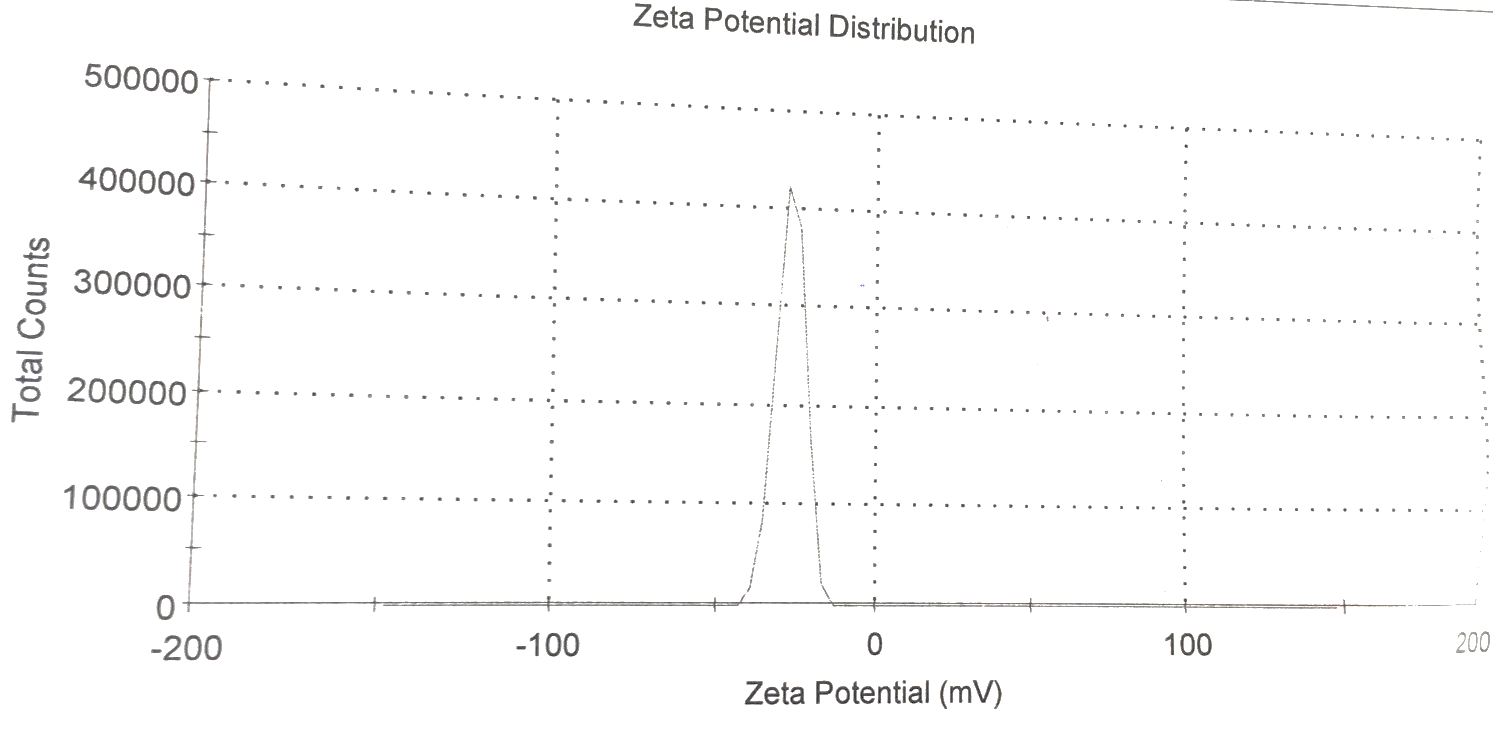


**Figure S1. DLS of Ti_3_C_2_Tx@Fe_3_O_4_@SiO_2_-FA**
